# Efficient Genomic Prediction of Yield and Dry Matter in Hybrid Potato

**DOI:** 10.3390/plants12142617

**Published:** 2023-07-11

**Authors:** James Adams, Michiel de Vries, Fred van Eeuwijk

**Affiliations:** 1 Biometris, Mathematical and Statistical Methods, Wageningen University and Research, 6708 PB Wageningen, The Netherlands; 2 Solynta, Dreijenlaan 2, 6703 HA Wageningen, The Netherlands

**Keywords:** hybrid potato, genomic prediction, general combining ability, genomically estimated breeding values, hybrid prediction, genomic prediction, genotype-by-environment

## Abstract

There is an ongoing endeavor within the potato breeding sector to rapidly adapt potato from a clonal polyploid crop to a diploid hybrid potato crop. While hybrid breeding allows for the efficient generation and selection of parental lines, it also increases breeding program complexity and results in longer breeding cycles. Over the past two decades, genomic prediction has revolutionized hybrid crop breeding through shorter breeding cycles, lower phenotyping costs, and better population improvement, resulting in increased genetic gains for genetically complex traits. In order to accelerate the genetic gains in hybrid potato, the proper implementation of genomic prediction is a crucial milestone in the rapid improvement of this crop. The authors of this paper set out to test genomic prediction in hybrid potato using current genotyped material with two alternative models: one model that predicts the general combining ability effects (GCA) and another which predicts both the general and specific combining ability effects (GCA+SCA). Using a training set comprising 769 hybrids and 456 genotyped parental lines, we found that reasonable a prediction accuracy could be achieved for most phenotypes with both zero common parents (ρ=0.36−0.61) and one (ρ=0.50−0.68) common parent between the training and test sets. There was no benefit with the inclusion of non-additive genetic effects in the GCA+SCA model despite SCA variance contributing between 9% and 19% of the total genetic variance. Genotype-by-environment interactions, while present, did not appear to affect the prediction accuracy, though prediction errors did vary across the trial’s targets. These results suggest that genomically estimated breeding values on parental lines are sufficient for hybrid yield prediction.

## 1. Introduction

Potato (*Solanum tuberosum* L.) is the most important starch source among vegetable crops. Cultivated for its edible tubers, potato’s end-use spans across multiple markets including table, chipping, frying, and industrial processing, with a global export value of USD 3.36 billion [1]. It has positive production characteristics including excellent water use efficiency and short crop cycles making it amenable to a variety of cropping systems [2]. Despite potato’s potential for food systems worldwide, slower genetic progress, particularly for quantitative traits such as yield, is a known impediment to long-term food security [3]. The underlying factors are numerous, but this is understood to be the consequence, in part, of potato’s polyploidy and large genetic load [4]. In the context of breeding, this makes potato an obligate outcrosser and affects the whole breeding system from initial crosses to the selection schemas used in identifying favorable varietal candidates. Together with potato’s large selection surface (as many as 40 traits), improving the quantitative traits in clonal potato is rife with technical difficulties [5,6].

There have been many proposed (and implemented) innovations for conventional clonal breeding including the use of marker-assisted selection, progeny testing strategies, and genetic transformation  [7]. Among these, one topic which has garnered significant interest over the past few decades is adapting the tetraploid potato (2n = 4x = 48) to a diploid hybrid breeding system (2n = 2x = 24) [8,9]. The appeal of diploid hybrid potato (DHP) breeding is understood to consist of benefits such as the use of true potato seed (TPS) directly in varietal evaluation [10], a segmented breeding program design [11], exploitation of heterotic vigor [12], and more efficient systems for germplasm production and dissemination [13]. With the gradual emergence of fertile diploid lines [14,15], research centered around diploid and hybrid potato breeding is gaining momentum across the world [9,16,17,18].

With all of hybrid breeding’s benefits, it is also known for being more resource and time intensive in contrast to inbred systems [19]. Hybrid breeding systems are also more complex in terms of breeding targets; not only must the breeder find the best set of parents (targeting general combining ability, i.e., GCA), but also find the best cross among that set of parents (targeting specific combining ability, i.e., SCA). Because of this, genomic prediction (GP) and genomic selection (GS) have become indispensable methods in the implementation of hybrid breeding in multiple crops [19,20]. The wide-spread adoption of these techniques has been made possible through the advent of high-throughput molecular marker data and the availability of powerful computational resources capable of rapid model fitting [21,22]. These methods are conceptual extensions of marker-assisted selection where rather than selecting an individual on its QTL status, selection is on the basis of genome-wide estimated breeding values (GEBVs). GP and GS have been used to increase the efficiency of hybrid breeding through better heterotic pool development [23], intra-population improvement [24], and even shortening breeding cycles by forgoing trialing altogether [25]. Because of GP’s and GS’s demonstrated gains in hybrid, and crop breeding more generally, developing appropriate models for each target trait with adequate training sets is a major pursuit in any breeding endeavor.

Recently, GP has received much attention in tetraploid potato with encouraging results demonstrated in chipping quality [26,27], disease resistance [28,29], fry color [30], dry matter content and specific gravity [26,31], yield components [29,31,32,33], and vine maturity [27,34]. Despite the increased interest in breeding DHP, there are currently no analogous studies confirming model performance for economically valuable traits. Additionally, while targeting the general combining ability (GCA) gives acceptable prediction accuracy in both simulation studies and in situ for many crops, evaluating the contribution of non-additive effects (often through specific combining ability, i.e., SCA) is a crucial question in GP’s implementation. The authors of this study wish to showcase GP in DHP with three primary aims. First, to demonstrate the feasibility of GP for the hybrid performance of multiple yield components and tuber dry matter content. Second, to test for the contribution of additive and non-additive genetic effects for each tuber phenotype. Lastly, a cursory examination on genotype-by-environment effects, to understand their impact on the predictions among the trials used in this study. Prediction models were constructed by adapting the modeling procedure of [35] by structuring the random genetic effects with marker information. Through this work, we touch upon several facets of predictive breeding, all of which are crucial for the adoption and improvement of the hybrid potato within the wider potato industry.

## 2. Materials and Methods

### 2.1. Populations and Trials

Between 2017 and 2020, several crossing blocks were constructed around 712 experimental inbred lines sourced from Solynta, a Dutch hybrid potato breeder [16]. These lines were generated from an original population comprising 16 founders and selected primarily based on the tuber criteria as well as on fertility characteristics during inbreeding. Across five nurseries, 1162 hybrids were produced through a sparse mating design, i.e., only a small fraction of crosses were conducted over the full crossing space ($n^{2}$) [36]. Hybrid TPS from these crosses were sown in trays in early March and transplanted in mid-May in both trial years. These hybrids were transplanted in all four field trials in two trial locations (Heelsum and Est, Netherlands) in 2019 and 2020. The trials conducted in 2019 tested the performance of 806 F1 crosses, as previously described in [35], with 356 additional hybrids evaluated in 2020 with a thin intersect of hybrids between years (43 hybrids) and thinner intersect across all trials (25 hybrids). All trials utilized a double ridge design comprising 16 plants per plot; ridges were 75 cm wide with plants spaced at 25 cm within the same ridge. Each trial utilized a randomized complete block design consisting of two replicates for each hybrid with additional control varieties planted in diagonals across blocks for spatial trend control. Haulm killing took place in September with subsequent harvests occurring two weeks later. All tubers, excluding those smaller than the 20 mm size class (due to instrumentation constraints), were harvested and measured on multiple traits including total tuber yield (TY; $Mg\cdot {\mathrm{Ha}}^{-1}$), total tuber number (TN; number of tubers per plot), percentage of tuber dry matter content (DM; percent), and average tuber volume (TV; cm^3^). All phenotyping procedures, trait definitions, and instrumentation used here are described in [37].

### 2.2. Marker Processing and Analysis

The parental lines used for test-crosses were genotyped using the targeted genotyping-by-sequencing (GBS) technology, SeqSNP^®^ [38]. These probes were designed around highly conserved regions across the genome. As per LGC’s protocol, leaf disks were sampled from each inbred line, placed in a 96-well plate, and sent to LGC for DNA extraction and analysis. Upon receipt of the results, the marker quality was assessed primarily on confirming the coverage and read depth for each sample. For the purpose of this study, these probes were filtered for high-quality biallelic SNPs using multiple criteria. These included typical filtering criteria such as minor allele frequency (p≥0.05) and percentage of SNP missingness (m≤0.1), along with polymorphism information content (PIC) and linkage disequilibrium (LD) between sites. The latter was chosen due to high LD between many SNPs resulting in redundant marker information. A SNP selection procedure was invoked using the $r_{v}^{2}$ metric provided by [39], which accounts for shared population history which tends to bias the traditional $r^{2}$ statistic. For any site in high LD with another ($r_{v}^{2}\geq 0.5$), the site with the highest PIC was chosen. This resulted in a filtered probe set containing 704 SNPs. There was a fraction of parental lines that were either genotyped with an irrelevant probe set or not genotyped at all. This resulted in 456 of the original 712 parental lines being present in the marker set.

**Figure 1:**
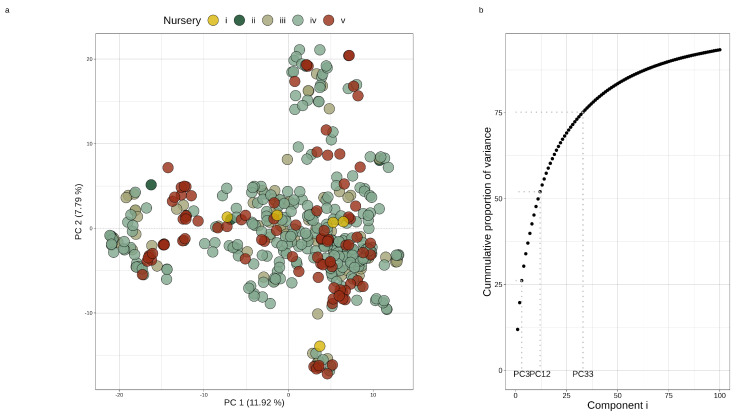
(**a**) Principal components 1 and 2 using filtered marker data (704 SNPs) for 456 parental lines. Parental lines are colored per nursery. (**b**) The corresponding scree plot showing the cumulative proportion of variance captured in the first 100 principal components with guides inserted where 25, 50, and 75 percent cumulative variance were captured (at components 3, 12, and 33, respectively).

Using this filtered marker set, a principal component analysis was applied to the marker data to assess any evident population structure. This was achieved by scaling the SNP matrix (X) with each SNP’s mean to produce a centered marker matrix, Z. R’s base svd() function was applied to Z to produce a matrix of left singular vectors and diagonal matrix of singular values (U and S, respectively) that were then used to derive the principal components of the original SNP covariance matrix (P=US). These components’ scores along with the covariance matrix’s eigenvalues were then used to assess the population structure (Figure 1).

Lastly, this scaled SNP matrix, Z, was used to compute several genomic relationship matrices. Additive genomic relationship matrices were computed for the 456 parents ($G_{p}$) as well as on the 769 hybrids ($G_{h}$) using VanRaden’s method ($G=\frac{{\mathrm{ZZ}}^{T}}{2\cdot p^{T}\left(1-p\right)}$) [40] via the R library AGHmatrix [41]. The dominance relationship matrix on hybrids was also estimated using the method proposed by [42]. Unless explicitly stated otherwise, all marker-based analysis was carried out using the R statistical language [43].

### 2.3. Statistical Analysis

A two-stage modeling approach was conducted to first model within each trial to produce unbiased estimates for each hybrid (BLUEs), which in turn were used in multi-year and location prediction models.

#### 2.3.1. First Stage

For the first stage, a model selection procedure was conducted on each trial and trait to account for specific field trends and spatial dynamics. Eleven different spatial models were utilized across all 16 trait and trial combinations. From these eleven spatial models, only four were selected based upon Akaike’s information criterion (AIC); these four spatial models are described in detail here. The base model equation has the form:

(1) $$y_{hb}=\mu +\alpha_{h}+\gamma_{b}+\varepsilon_{hb}$$

where the phenotype, *y*, is modeled on hybrid *h* (α) and block *b* (γ), both effects being treated as fixed. A residual, ε, was modeled as random but its variance structure varied per spatial model chosen. There was a modification of this base model structure where a random column effect was included:

(2) $$y_{hbm}=\mu +\alpha_{h}+\gamma_{b}+c_{m}+\varepsilon_{hbm}$$

with an additional random effect ($c∼N(0,\sigma_{c}^{2})$) on the plot in column *m* (*c*) in the trial field. There were four residual variance structures among the several spatial models chosen. The simplest was a residual variance with an independent and identically distributed (IID) Gaussian distribution such that:

(3) $$\mathrm{var}\left(\varepsilon \right)=\sigma_{e}^{2}I$$

That is, the residual is modeled with a constant spatial variance ($\sigma_{e}^{2}$) which is invariant regardless of a plot’s position in the field. The second variance structure utilizes a 1st order autoregressive structure along the column coordinates. This can be expressed as:

(4) $$\mathrm{var}\left(\varepsilon \right)=\sigma_{e}^{2}I_{r}⊗\Sigma_{c}\left(\rho_{c}\right)$$

where the residual variance is modeled as the Kronecker product of an identity matrix ($I_{r}$: each dimension is equal to the number of rows in the trial field) and a 1st order autoregressive matrix ($\Sigma_{c}$: each dimension is equal to the number of columns in the trial field) scaled by the spatial variance, $\sigma_{e}^{2}$. The autoregressive matrix, $\Sigma_{c}$, is parameterized by the autocorrelation parameter, $\rho_{c}$, so that the covariances decline exponentially (i.e., $\Sigma_{c_{ij}}=\rho_{c}^{\left|i-j\right|}$). The third residual structure builds on this with the addition of a spatially-independent error, often referred to as a nugget effect. This results in:

(5) $$\mathrm{var}\left(\varepsilon \right)=\sigma_{e}^{2}I_{r}⊗\Sigma_{c}\left(\rho_{c}\right)+\sigma_{\eta }^{2}$$

where the spatially-independent variance, $\sigma_{\eta }^{2}$, is the error variance not attributable to the spatial components, traditionally attributed to harvest- and instrument-related error [44]. The last spatial error builds on the model (5) with the addition of an autoregressive effect along the rows. This is then:

(6) $$\mathrm{var}\left(\varepsilon \right)=\sigma_{e}^{2}\Sigma_{r}\left(\rho_{r}\right)⊗\Sigma_{c}\left(\rho_{c}\right)+\sigma_{\eta }^{2}$$

where $\Sigma_{r}$ is a 1st order autoregressive matrix ($\Sigma_{r}$: rank is same as the number of rows in the trial field) and defined by the autocorrelation parameter, $\rho_{r}$. The combination of the base model and residual variance structure is given in Table 1 for each trait and trial combination. BLUEs were then produced from each best supported spatial model and used for all genetic model training going forward.

#### 2.3.2. Phenotypic Variance and Stability

To evaluate the genetic variance of the hybrids across trials, raw data from each trial was used to create the following multi-trial model:

(7) $$y_{bfht}=\beta_{f}+\gamma_{fb}+a_{ht}+\varepsilon_{bfht}$$

where the hybrid response (*y*) is modeled on a fixed trial intercept (β) for the trial, *f*, a fixed within-trial block effect (γ) for block *b* in trial *f*, a random hybrid by location interaction (*a*) for hybrid *h* in location *t*, and an IID Gaussian error term, ε ($\varepsilon ∼N\left(0,\sigma_{\varepsilon }^{2}\right)$). The hybrid by location effect was modeled with an unstructured covariance structure so that there was a separate hybrid variance for each trial location along with their covariance. This is described by:

$$a∼N\left(0,\Sigma_{a}\right)\;\;\;\;\;\;\;\;\;\;\;\;\;\Sigma_{a}=\left[\begin{array}{cc} \sigma_{G(\mathrm{Est})}^{2} & \sigma_{G(\mathrm{Est}),G(\mathrm{Hee})}\\ \sigma_{G(\mathrm{Hee}),G(\mathrm{Est})} & \sigma_{G(\mathrm{Hee})}^{2} \end{array}\right]$$

Using model (7), across-trial BLUPs were predicted for each hybrid. Several variance ratios were also produced from this model including broad-sense heritabilities ($H^{2}=\frac{\sigma_{a}^{2}}{\sigma_{a}^{2}+\sigma_{\varepsilon }^{2}}$) per trial location and inter-genotype coefficients of variation (${\mathrm{CV}}_{G}=\frac{\sigma_{a}}{\mu }$) using the variances embedded within $\Sigma_{a}$ and means for each respective trial location. The intra-genotype coefficient of variation (${\mathrm{CV}}_{\varepsilon }=\frac{\sigma_{\varepsilon }}{\mu }$) was also computed using the residual variance and global mean. These variance ratios are reported in Table 2 for each tuber phenotype. All models used for the first-stage and phenotypic models were fit, examined, and tested using ASReml-R version 4, provided by VSN International [45].

#### 2.3.3. Second Stage

Hybrid BLUEs from each trial were treated as the phenotypic response for second-stage models. BLUEs were carried along with their respective standard errors to properly weight each observation by its precision. This was achieved using a diagonal structure on the squared standard errors for each trial *f* to approximate the full residual covariance structure ($R={⨁}_{f=1}^{t}R_{f}$ with $R_{f}=\mathrm{Diag}\left({\hat{s}}_{f}^{2}\right)$) [46,47]. These second-stage models take the form of multi-environment models with two parameterizations for the hybrid genetic variance. Because we are interested in measuring the benefits of dominance genetic variance in genomic prediction, one model bases its prediction solely on parental general combining ability effects (GCA model) and the second on both general and specific combining ability effects (GCA+SCA model). The GCA model is:

(8) $${\hat{\mu }}_{ijf}=\beta_{f}+g_{i}+g_{j}+\delta_{ij}+t_{ijf}+r_{ijf}$$

where $\hat{\mu }$, the hybrid BLUE, is modeled on a fixed trial intercept (β) for trial *f*, the GCA (g) of parents *i* and *j*, a hybrid by trial interaction (*t*) with trial *f* and hybrid ij, a genetic residual term (δ) for hybrid ij, and a residual, *r*. All random effects have Gaussian distributions centered about 0 with the variances of GCA and hybrid-trial interactions being structured by the genomic relationship matrices $G_{p}$ and $G_{h}$, respectively. GCA variance was modeled as a scaling of the additive parental genomic relationship matrix ($\sigma_{gca}^{2}G_{p}$) while the hybrid by trial variance was represented by the Kronecker product of an identity matrix containing the GCA by trial interaction variance and the hybrid additive genomic relationship matrix ($\sigma_{gxt}^{2}I_{t}⊗G_{h}$). The variance of the genetic residual was modeled as IID so that the total hybrid variance according to the GCA model is $2\sigma_{gca}^{2}Z_{p}G_{p}Z_{p}^{t}+\sigma_{\delta }^{2}I$, where $Z_{p}$ is the incidence matrix assigning parent lines to hybrid crosses. The residual variance is considered to be known (estimated in stage 1) so that the full residual distribution is $r_{ijf}∼N\left(0,R_{ijf}\right)$. The GCA+SCA model differs from the GCA model in the addition of the SCA in the hybrid, making the full model:

(9) $${\hat{\mu }}_{ijf}=\beta_{f}+g_{i}+g_{j}+s_{ij}+\delta_{ij}+t_{ijf}+r_{ijf}$$

where *s* is the specific combining ability for parents *i* and *j*. The variance of the SCA effect is defined by the dominance relationship matrix scaled by a scalar variance component ($\sigma_{sca}^{2}D$) making the hybrid genetic variance in the GCA+SCA model equal to $2\sigma_{gca}^{2}Z_{p}G_{p}Z_{p}^{t}+\sigma_{sca}^{2}D+\sigma_{\delta }^{2}I$. The modeling procedure of [31] was adapted by estimating the $\sigma_{gca}^{2}$ and $\sigma_{gxt}^{2}$ parameters in the GCA model followed by fixing these same components in the GCA+SCA models to these estimates. These model constraints allowed for the partitioning of the GCA and SCA variance. Having said this, the genetic residual term, in addition to containing higher-order interactions, is not orthogonal with the SCA term. This is partially reflected in deviations in total genetic variance between the GCA and GCA+SCA models (Table 3).

### 2.4. Model Testing

**Figure 2:**
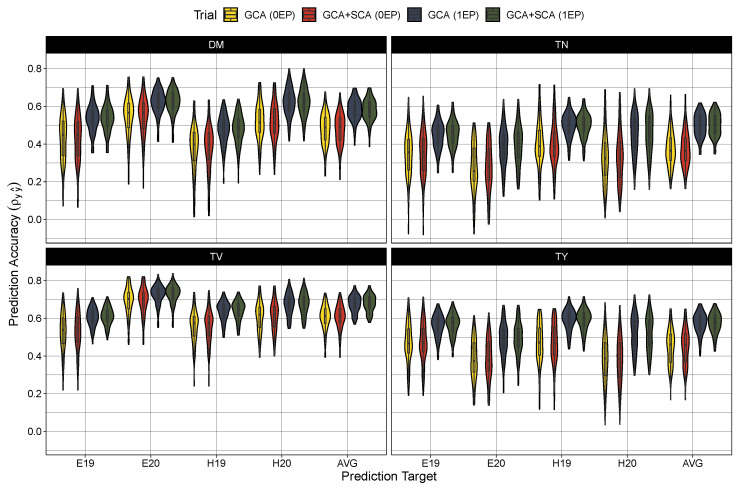
Predictive accuracy (correlation coefficient of true hybrid performance and predicted cross value) in the zero evaluated parent set (0EP) and the one evaluated parent set (1EP) for the GCA and GCA+SCA models for dry matter content (DM), total tuber number (TN), mean tuber volume (TV), and total tuber yield (TY). Each trait, model, and testing scenario were tested 100 times. Predictions were either compared to BLUEs generated from the first stage of modeling for Est 2019 and 2020 (E19 and E20, respectively) and Heelsum 2019 and 2020 (H19 and H20, respectively) or against across-trial BLUPs (AVG) produced from the Model (7).

**Figure 3:**
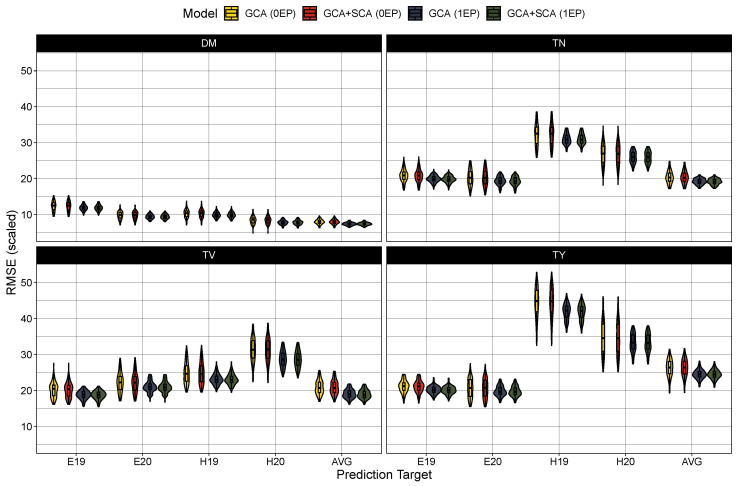
Scaled root mean square error ($100\cdot \sqrt{E{\left(\hat{y}-y\right)}^{2}}/\mu$) in prediction in the zero common parent (0EP) and one common parent (1EP) sets for the GCA and GCA+SCA models for dry matter content (DM), total tuber number (TN), mean tuber volume (TV), and total tuber yield (TY). Each trait, model, and testing scenario were tested 100 times. Predictions were either compared to BLUEs generated from the first stage of modeling for Est 2019 and 2020 (E19 and E20, respectively) and Heelsum 2019 and 2020 (H19 and H20, respectively) or against across-trial BLUPs (AVG) produced from Model (7).

To test the advantage of including dominance effects in GP for hybrid potato, we examine the performance of the GCA (8) and GCA+SCA (9) models. The authors define the model performance using the prediction accuracy and error on a given test set. The prediction accuracy was assessed using Pearson’s correlation coefficient between the true and predicted hybrid performance ($\rho_{y,\hat{y}}=\frac{\mathrm{cov}(y,\hat{y})}{\mathrm{sd}\left(y\right)\cdot \mathrm{sd}\left(\hat{y}\right)}$) and prediction error by the root mean square error scaled by the trait mean for interpretive convenience (scaled RMSE $=100\cdot \frac{\sqrt{E({\left(y-\hat{y}\right)}^{2})}}{\mu }$). The testing and training strategy was conducted by defining two test sets with different levels of parental information shared between training and test sets [48]. This procedure begins by randomly assigning all hybrid parents into an evaluated and unevaluated set. Consequently, these parental groups randomly assign hybrids to three sets, that is, hybrids with zero evaluated parents (0EP), hybrids with one evaluated parent (1EP), and hybrids with two evaluated parents (the training set). This allows for model testing which mirrors breeding scenarios where a hybrid is produced from a test cross of elite and novel parents (1EP) and crossing of two novel parents (0EP). Because one of the aims of this study was evaluating the prediction of genotype by trial effects, we define true hybrid performance as (1) the hybrid average performance across trials (AVG), and (2) performance within individual trials (E19, E20, H19, H20). This testing scheme was repeated 100 times for each tuber phenotype and genetic model to assess model accuracy (Figure 2) and error (Figure 3) under multiple scenarios. Because the absorption of coefficient matrices failed using ASReml-R, model fitting and cross-validation for the second-stage models (Equations (8) and (9)) were instead performed using the sommer R package (version 4.1.6), a multivariate mixed model solver, using its average information method [49].

## 3. Results

### 3.1. Trial and Phenotype Analysis

Among all 16 trial and trait combinations, the majority were best fit with both an autoregressive trend and nugget effect (Table 1). However, a variant of a traditional RCBD design was selected for dry matter content in Heelsum 2019. The severity of the spatial heterogeneity varied per trait in each trial, but spatial trends in the Est trial field in 2019 were large for all phenotypes. The spatial model which assumed an IID residual (i.e., dry matter content in Heelsum 2019) had empirical semivariograms which were relatively stable with the exception of the last 60 observations, consistent with an edge effect near the end of the trial field.

Using the variance components from Model (7), multiple variance ratios were produced for all tuber phenotypes (Table 2). Broad-sense heritabilities ranged between 0.47–0.73 in Est and 0.55–0.82 in Heelsum, with average tuber volume being the most heritable trait over both locations. The heritabilities between Est and Heelsum were most divergent for total yield (0.47 and 0.81, respectively) and tuber number (0.49 and 0.73, respectively). This coincides with the genetic coefficients of variation which exhibited greater hybrid variation in Heelsum (between 27% and 35%) than the Est trial field (between 23% and 28%) for all traits but dry matter content. Dry matter content showed a larger genetic variance in Est over Heelsum ($H^{2}$ of 0.68 versus 0.55), in contrast to what was seen in the other phenotypes. This could be due to a positive bias in tuber dry matter content among smaller tubers which were more prevalent under the clay conditions of the Est field trial. The error coefficient of variation was similar among all yield phenotypes (between 14% in average tuber volume and 22% in total tuber yield), with dry matter content having the smallest percentage of error (7.9%). The small coefficients of variation seen in dry matter content (relative to other traits) are thought to be artefactual due to its scale. The maximum variance that can be observed in dry matter content is likely smaller due to the very tight bounds around biologically relevant dry matter values (see Bhatia and Davis [50]).

### 3.2. Marker Analysis

The filtered SNP dataset was used for population analysis and the construction of both additive genomic relationship matrices ($G_{p}$ and $G_{h}$) and the dominance relationship matrix for hybrids (D). The principal component analysis revealed little evident population structure aside from a few minor family clusters (Figure 1a). Less than 20% of the original marker covariance was captured on the first two principal axes, with 75% of its variance distributed across the first 33 axes (Figure 1b). Along with this, there was no clear relationship between the older and newer nurseries and the first or second principal loadings, suggesting little differentiation between these different sub-populations over time. This analysis was complemented with the Bayesian population software, STRUCTURE, to determine the number of ancestral founders that gave rise to this population [51]. This analysis found no evidence of recent admixture events, with one ancestral population being the most probable origin for this panel of inbred lines. Both findings suggest little population structure in this panel needing to be taken into account whilst modeling.

### 3.3. Genetic Modelling and Genomic Prediction

Both the GCA (8) and GCA+SCA (9) sets of models converged for all traits studied here. Credible variance components were also extracted for all eight models (Table 3). GCA variance was the largest variance component for all traits except total tuber yield where GCA variance was nearly equal with the hybrid by trial interaction component (TY: $\sigma_{gca}^{2}=7.4$, $\sigma_{gxt}^{2}=7.8$). With respect to total genetic variance, the additive variance was the largest genetic effect for all phenotypes (between 63% and 84%). The proportion of SCA genetic variance in the full GCA+SCA models was noticeably smaller in dry matter content (9%) in contrast to the other yield components (14–17%). It is worth noting that the genetic residual term in both the GCA and GCA+SCA models contained a considerable portion of genetic variance. This was most apparent in both the GCA and GCA+SCA models for tuber number (32% and 20%, respectively), though present in total tuber yield (26% and 17%, respectively) and average tuber volume (24% and 13%, respectively) as well.

Turning to model performance, there were no measurable differences between the GCA and GCA+SCA models in predicting the average hybrid performance (Table 4) or among any of the trial-specific prediction targets (Figure 2). As expected, prediction accuracy was globally deflated in the 0EP set ($\rho_{y,\hat{y}}$ between 0.36 and 0.61) in contrast to the 1EP set ($\rho_{y,\hat{y}}$ between 0.50 and 0.68) (Table 4). These coincided with larger root mean square errors in the 0EP set (scaled RMSE = 8–26.4%) over the 1EP set (scaled RMSE = 7.4–24.5%). Looking among the traits, the prediction accuracy was highest for average tuber volume (0EP: 0.61 and 1EP: 0.68), followed by dry matter content (0EP: 0.49 and 1EP: 0.58) and total tuber yield (0EP: 0.46 and 1EP: 0.58), with considerably lower prediction error in the models for dry matter (7.4–8.0%) than for average tuber volume (18.9–20.7%) and total tuber yield (24.5–26.4%). Tuber number had the lowest accuracy among the traits ($\rho_{y,\hat{y}}$ between 0.36 and 0.51), with intermediate prediction errors (18.9–20.7%). When examining the performance among the trial-specific targets, prediction accuracy did vary per trait and trial-target. In general, prediction accuracy tended to be lower among the 2019 trial sets (i.e. E19 and H19) for dry matter content and average tuber volume while accuracy was lower in the 2020 trial sets (i.e., E20 and H20) for tuber number and total tuber yield. These differences in performance between trial targets, however, become much more apparent when examining the prediction error (Figure 3). The root mean square error was largest (and most variable among prediction targets) in total yield, and to a lesser extent, tuber number, with errors being highest for the Heelsum trial sets (i.e., H19 and H20) in both phenotypes. Average tuber volume also exhibited a higher RMSE for predictions in the Heelsum 2020 target while the RMSEs in dry matter content were much smaller and relatively uniform across trait targets.

## 4. Discussion

Hybrid breeding has been revolutionized by GS and GP, making adoption of these techniques a key step in accelerating quantitative improvement. Of particular importance when implementing these technologies is determining the contribution and nature of the genetic effects in the relevant target and then assessing how molecular marker and pedigree information can best provide predictive power for it. This in turn provides a roadmap for breeders to place these predictive methods where they can be most efficiently applied. Additionally, understanding the presence of genotype-by-environment interactions has also received increasing attention as selection continues to shift away from general performance to adaptability to specific envirotypes. Our findings represent a first inspection of GP for multiple tuber traits in hybrid potato using conventional genetic models.

### 4.1. Feasibility of Genomic Prediction in Hybrid Potato

GP was possible in both testing scenarios (0EP and 1EP) in both genetic models (GCA and GCA+SCA) for all tuber traits, though not equally among them. Average tuber volume had the highest prediction accuracy in both test scenarios (0.61 and 0.68, respectively) followed by dry matter content and total tuber yield. Looking at other available studies, dry matter content (and starch) has a consistently high prediction accuracy (0.73–0.84) in tetraploid populations [26,29,33]. Total yield (and components of yield) is more variable (0.31–0.66), reflecting different modeling strategies and training set properties [29,32]. Among our traits studied, total tuber number exhibited the poorest prediction accuracy, especially in the 0EP testing scenario (Table 4). [32] similarly found relatively poor model performance for tuber number, with their additive model showing an average accuracy of 0.35. In contrast to the tetraploid studies, our model accuracies were notably lower. This can likely be attributed to differences in testing strategies and a smaller training to test set ratio (expectation of 1 and 0.5 for the 0EP and 1EP sets, respectively) resulting in a more severe testing in contrast to the k-fold cross-validation strategy invoked elsewhere.

### 4.2. Contribution of Non-Additive Effects in Prediction

We found no practical benefit in the incorporation of dominance effects (via SCA) for the GP model’s performance in any tuber phenotype. The lack of added value with SCA also appears inconsistent given the magnitude of SCA variances measured here (Table 3) and in our preceding phenotypic study [35]. As highlighted there, several lines of reasoning can be pursued while considering non-additive genetic effects. Reflecting on the sparse crossing design used to generate these hybrids, its not unexpected for shrinkage of the SCA effects even if the model is technically identifiable. If this is the case, then larger training sets would be required for exploitation of SCA among hybrids. Alternatively, reflecting on the size of the genetic residual (between 20 and 37% of $\sigma_{G}^{2}$), kernel-based approaches could be more informative in predicting hybrid performance [52,53]. Rather than decomposing the total genetic value into separate additive and non-additive components, prediction is instead based upon the total observed genetic value [54]. The application of such semi-parametric approaches could be an interesting avenue for future prediction modeling in DHP.

### 4.3. Hybrid Prediction and Genotype-by-Environment Interactions

While present, genotype-by-environment interactions did not appear to affect model performance for dry matter content and average tuber volume. This coincides with relatively consistent heritabilities between trial locations and the small $\sigma_{gxt}^{2}$ variance components for both traits. Several studies in diploid [37] and tetraploid [29,31,33,55] potato also corroborate the high stability of dry matter content. Examining total yield and tuber number, prediction accuracy appears fairly stable across target environments, but prediction errors were highly dependent on the target environment (Figure 3). This was especially apparent between the Heelsum and Est 2019 trials, where the difference in median scaled RMSE was over 25% and 12%, respectively. Considering the significant differences in heritabilities between trial locations (Table 2), this could negatively impact prediction accuracy if the genotype-by-environment structure does not reflect this. Similar to what has been reported in tetraploid potato [29,33,55], the total yield is highly influenced by genotype-by-environment interactions and difficult to structure. For future work in genotype-by-environment interactions, designing trials around known abiotic stresses in potato would allow for more meaningful decomposition of these effects. Doing so in climates which target relevant conditions for the world’s potato growers is also important in a GP context for the prediction of hybrid adaptability.

### 4.4. Genomic Prediction for Breeders

These results have some implications for potato breeders. First, this study gives ample evidence that stable additive genetic effects are not only able to be estimated for potato inbred lines, but can also be leveraged for the prediction of a parent’s breeding value in a dedicated cross. In particular, these results show the potential of genomic prediction in both a test cross and novel crossing set context through the use of the 1EP and 0EP test sets, respectively. Based upon these results, confident predictions can be offered in both contexts for all phenotypes studied here with the exception of total tuber number, which exhibited depressed model accuracy, making it too unreliable in a novel crossing set for breeders. The GCA variance being the largest genetic effect among all traits is also worth reiterating as it directs breeders towards prioritizing additive variance among multiple tuber traits. This directly touches upon a breeding program’s structure and how it exploits additive variation during population improvement among highly heritable traits.

## 5. Conclusions

In this study, we have laid the groundwork for genomic prediction in hybrid potato with simple genetic models and no training set optimization. We demonstrate the GEBVs estimated from an additive model (our GCA model) suffices in predicting hybrid potato performance in dry matter content, average tuber volume, and total yield. The effect of genotype-by-environment interactions is mostly relevant for yield components such as tuber number and total yield, while dry matter content and average tuber volume were remarkably stable among target environments for genomic prediction. Principally, these results show for the first time that genomic prediction can be leveraged for inbred and hybrid selection in diploid hybrid potato. The adoption of predictive breeding in diploid potato is invaluable for the continued genetic progress of this new hybrid crop.

## Figures and Tables

**Table 1:** Selected spatial models across four field trials for percentage dry matter content (DM), total tuber number (TN), average tuber volume (TV), and total tuber yield (TY). Each entry is composed of the base model along with the variance structure of the residual.

Trait	Est Field Trial		Heelsum Field Trial	
	2019	2020	2019	2020
DM	(1) + (6)	(1) + (6)	(2) + (3)	(1) + (5)
TN	(1) + (6)	(1) + (6)	(1) + (6)	(1) + (6)
TV	(1) + (6)	(1) + (4)	(1) + (6)	(1) + (6)
TY	(1) + (6)	(1) + (6)	(1) + (6)	(1) + (6)

**Table 2:** Variance ratios from multi-trial model for percentage dry matter content (DM), total tuber number (TN), average tuber volume (TV), and total tuber yield (TY). The ratios include broad-sense heritabilities observed in Est and Heelsum (${H_{\mathrm{Est}}}^{2}$ and ${H_{\mathrm{Hee}}}^{2}$), the inter-genotype coefficients of variation from Est and Heelsum (${\mathrm{CV}}_{G:\mathrm{Est}}$ and ${\mathrm{CV}}_{G:\mathrm{Hee}}$), and the intra-genotype coefficients of variation ${\mathrm{CV}}_{\varepsilon }$.

	DM	TN	TV	TY
$H_{\mathrm{Est}}^{2}$	0.68	0.49	0.73	0.47
$H_{\mathrm{Hee}}^{2}$	0.55	0.73	0.82	0.81
${\mathrm{CV}}_{G(\mathrm{Est})}$	11.06	23.57	24.82	28.33
${\mathrm{CV}}_{G(\mathrm{Hee})}$	9.19	27.77	27.81	35.91
${\mathrm{CV}}_{\varepsilon }$	7.91	19.74	14.00	22.11

**Table 3:** Variance components for the GCA and GCA+SCA genetic models for percentage dry matter content (DM), total tuber number (TN), average tuber volume (TV), and total tuber yield (TY). Included are the variances attributed to general combining ability, hybrid by trial interaction, specific combining ability, genetic residual, and the proportions of genetic variance among each genetic component.

Trait	Variance Components:	$\sigma_{gca}^{2}$	$\sigma_{gxt}^{2}$	$\sigma_{sca}^{2}$	$\sigma_{\delta }^{2}$	$\frac{2\sigma_{gca}^{2}}{\sigma_{G}^{2}}$	$\frac{\sigma_{sca}^{2}}{\sigma_{G}^{2}}$	$\frac{\sigma_{\delta }^{2}}{\sigma_{G}^{2}}$
DM	GCA	1.1	0.5		0.4	0.84		0.16
	GCA+SCA			0.3	0.3	0.80	0.09	0.11
TN	GCA	368.9	287.7		341.0	0.68		0.32
	GCA+SCA			195.2	232.2	0.63	0.17	0.20
TV	GCA	11.8	2.7		7.2	0.76		0.24
	GCA+SCA			5.5	4.4	0.71	0.16	0.13
TY	GCA	7.4	7.8		5.3	0.74		0.26
	GCA+SCA			3.0	3.6	0.69	0.14	0.17

**Table 4:** Median prediction accuracy and median scaled root mean square error for each trait, genetic model, and test set. This was conducted on dry matter content (DM), total tuber number (TN), mean tuber volume (TV), and total tuber yield (TY). Predictions were tested against average hybrid performance (AVG) produced from Model (7).

Phenotype	0 Evaluated Parents Set		1 Evaluated Parent Set	
	GCA	GCA+SCA	GCA	GCA+SCA
DM	0.49 (8.0)	0.49 (8.0)	0.58 (7.4)	0.58 (7.4)
TN	0.36 (20.3)	0.36 (20.2)	0.50 (19.2)	0.51 (19.2)
TV	0.61 (20.7)	0.61 (20.7)	0.68 (19.0)	0.68 (18.9)
TY	0.46 (26.4)	0.46 (26.4)	0.58 (24.5)	0.58 (24.5)

## Data Availability

Data used for the production of these findings are available upon request. Restrictions apply to the availability of specific data sets. Data related queries can be made to James Adams at james.adams@wur.nl with the permission of Solynta Hybrid Breeding.

## References

[1] (2023). FAO.STAT. Food and Agriculture Organization of the United Natons. http://www.fao.org/faostat..

[2] Haverkort A.J. (1990). Ecology of potato cropping systems in relation to latitude and altitude. Agric. Syst..

[3] Douches D., Maas D., Science K.J.C. (1996). Assessment of potato breeding progress in the USA over the last century. Crop Sci..

[4] Lian Q., Tang D., Bai Z., Qi J., Lu F., Huang S., Zhang C. (2019). Acquisition of deleterious mutations during potato polyploidization. J. Integr. Plant Biol..

[5] Gebhardt C. (2013). Bridging the gap between genome analysis and precision breeding in potato. Trends Genet..

[6] Gopal J. (2015). Challenges and Way-forward in Selection of Superior Parents, Crosses and Clones in Potato Breeding. Potato Res..

[7] Bradshaw J.E. (2022). A Brief History of the Impact of Potato Genetics on the Breeding of Tetraploid Potato Cultivars for Tuber Propagation. Potato Res..

[8] Lindhout P., Meijer D., Schotte T., Hutten R.C.B., Visser R.G.F., Van Eck H.J., Lindhout P., Meijer D., Hutten R.C.B., Visser R.G.F. (2011). Towards F 1 Hybrid Seed Potato Breeding. Potato Res..

[9] Jansky S.H., Charkowski A.O., Douches D.S., Gusmini G., Richael C., Bethke P.C., Spooner D.M., Novy R.G., De Jong H., De Jong W.S. (2016). Reinventing potato as a diploid inbred line-based crop. Crop Sci..

[10] Van Dijk L., de Vries M., Lommen W., Struik P. (2021). Transplanting hybrid potato seedlings at increased densities enhances tuber yield and shifts tuber-size distributions. Potato Res..

[11] Technow F., Podlich D., Cooper M. (2021). Back to the future: Implications of genetic complexity for the structure of hybrid breeding programs. G3 Genes Genomes Genet..

[12] Technow F., Riedelsheimer C., Schrag T.A., Melchinger A.E. (2012). Genomic prediction of hybrid performance in maize with models incorporating dominance and population specific marker effects. Theor. Appl. Genet..

[13] Pallais N. (1991). True Potato Seed: Changing Potato Propagation from Vegetative to Sexual. HortScience.

[14] Eggers E.J., van der Burgt A., van Heusden S.A.W., de Vries M.E., Visser R.G.F., Bachem C.W.B., Lindhout P. (2021). Neofunctionalisation of the Sli gene leads to self-compatibility and facilitates precision breeding in potato. Nat. Commun..

[15] Ma L., Zhang C., Zhang B., Tang F., Li F., Liao Q., Tang D., Peng Z., Jia Y., Gao M. (2021). A nonS-locus F-box gene breaks self-incompatibility in diploid potatoes. Nat. Commun..

[16] Lindhout P., de Vries M., ter Maat M., Ying S., Marcela V.-Z., van Deusden S., Gefu W.-P. (2018). Hybrid potato breeding for improved varieties. Achieving Sustainable Cultivation of Potatoes Volume 1.

[17] Bethke P.C., Halterman D.A., Francis D.M., Jiang J., Douches D.S., Charkowski A.O., Parsons J. (2022). Diploid Potatoes as a Catalyst for Change in the Potato Industry. Am. J. Potato Res..

[18] Zhang C., Yang Z., Tang D., Zhu Y., Wang P., Li D., Zhu G., Xiong X., Shang Y., Li C. (2021). Genome design of hybrid potato. Cell.

[19] Labroo M.R., Studer A.J., Rutkoski J.E. (2021). Heterosis and Hybrid Crop Breeding: A Multidisciplinary Review. Front. Genet..

[20] Zhao Y., Mette M.F., Reif J.C. (2015). Genomic selection in hybrid breeding. Plant Breed..

[21] Meuwissen T.H.E., Hayes B.J., Goddard M.E. (2001). Prediction of Total Genetic Value Using Genome-Wide Dense Marker Maps. Genetics.

[22] Bernardo R. (2016). Bandwagons I, too, have known. Theor. Appl. Genet..

[23] Rembe M., Zhao Y., Jiang Y., Reif J.C. (2019). Reciprocal recurrent genomic selection: An attractive tool to leverage hybrid wheat breeding. Theor. Appl. Genet..

[24] Gaynor R.C., Gorjanc G., Bentley A.R., Ober E.S., Howell P., Jackson R., Mackay I.J., Hickey J.M. (2017). A Two-Part Strategy for Using Genomic Selection to Develop Inbred Lines. Crop Sci..

[25] Heffner E.L., Lorenz A.J., Jannink J.L., Sorrells M.E. (2010). Plant Breeding with Genomic Selection: Gain per Unit Time and Cost. Crop Sci..

[26] Sverrisdóttir E., Sundmark E.H.R., Johnsen H.∅., Kirk H.G., Asp T., Janss L., Bryan G., Nielsen K.L. (2018). The Value of Expanding the Training Population to Improve Genomic Selection Models in Tetraploid Potato. Front. Plant Sci..

[27] Pandey J., Scheuring D.C., Koym J.W., Endelman J.B., Vales M.I. (2022). Genomic selection and genome-wide association studies in tetraploid chipping potatoes. The Plant Genome.

[28] Enciso-Rodriguez F., Douches D., Lopez-Cruz M., Coombs J., de los Campos G. (2018). Genomic Selection for Late Blight and Common Scab Resistance in Tetraploid Potato (Solanum tuberosum). G3 Genes Genomes Genet..

[29] Ortiz R., Crossa J., Reslow F., Perez-Rodriguez P., Cuevas J. (2022). Genome-Based Genotype × Environment Prediction Enhances Potato (*Solanum tuberosum* L.) Improvement Using Pseudo-Diploid and Polysomic Tetraploid Modeling. Front. Plant Sci..

[30] Byrne S., Meade F., Mesiti F., Griffin D., Kennedy C., Milbourne D. (2020). Genome-Wide Association and Genomic Prediction for Fry Color in Potato. Agronomy.

[31] Endelman J.B., Carley C.A.S., Bethke P.C., Coombs J.J., Clough M.E., da Silva W.L., De Jong W.S., Douches D.S., Frederick C.M., Haynes K.G. (2018). Genetic Variance Partitioning and Genome-Wide Prediction with Allele Dosage Information in Autotetraploid Potato. Genetics.

[32] Wilson S., Zheng C., Maliepaard C., Mulder H.A., Visser R.G.F., van der Burgt A., van Eeuwijk F. (2021). Understanding the Effectiveness of Genomic Prediction in Tetraploid Potato. Front. Plant Sci..

[33] Cuevas J., Reslow F., Crossa J., Ortiz R. (2022). Modeling genotype × environment interaction for single- and multi-trait genomic prediction in potato (*Solanum tuberosum* L.). Plant Biol..

[34] Slater A.T., Cogan N.O., Forster J.W., Hayes B.J., Daetwyler H.D. (2016). Improving Genetic Gain with Genomic Selection in Autotetraploid Potato. Plant Genome.

[35] Adams J.R., de Vries M.E., Zheng C., van Eeuwijk F.A. (2022). Little heterosis found in diploid hybrid potato: The genetic underpinnings of a new hybrid crop. G3 Genes Genomes Genet..

[36] Wang X., Zhang Z., Xu Y., Li P., Zhang X., Xu C. (2020). Using genomic data to improve the estimation of general combining ability based on sparse partial diallel cross designs in maize. Crop J..

[37] Stockem J., de Vries M., van Nieuwenhuizen E., Lindhout P., Struik P.C. (2020). Contribution and Stability of Yield Components of Diploid Hybrid Potato. Potato Res..

[38] LGC (2019). SeqSNP Targeted GBS as Alternative for Array Genotyping in Routine Breeding Programs [White Paper]. https://biosearch-cdn.azureedge.net/assetsv6/seqsnp-tgbs-alternative-genotyping-routine-breeding-programs.pdf.

[39] Mangin B., Siberchicot A., Nicolas S., Doligez A., This P., Cierco-Ayrolles C. (2012). Novel measures of linkage disequilibrium that correct the bias due to population structure and relatedness. Heredity.

[40] VanRaden P.M. (2008). Efficient methods to compute genomic predictions. J. Dairy Sci..

[41] Amadeu R.R., Cellon C., Olmstead J.W., Garcia A.A.F., Resende M.F.R., Muñoz P.R. (2016). AGHmatrix: R Package to Construct Relationship Matrices for Autotetraploid and Diploid Species: A Blueberry Example. Plant Genome.

[42] Su G., Christensen O.F., Ostersen T., Henryon M., Lund M.S. (2012). Estimating Additive and Non-Additive Genetic Variances and Predicting Genetic Merits Using Genome-Wide Dense Single Nucleotide Polymorphism Markers. PLoS ONE.

[43] R Core Team (2022). R: A Language and Environment for Statistical Computing. https://www.R-project.org/.

[44] Müller B.U., Kleinknecht K., Möhring J., Piepho H.P. (2010). Comparison of spatial models for sugar beet and barley trials. Crop Sci..

[45] Butler D.G., Cullis B.R., Gilmour A.R., Gogel B.J., Thompson R. (2017). ASReml-R Reference Manual Version 4.

[46] Frensham A., Cullis B., Verbyla A. (1997). Genotype by Environment Variance Heterogeneity in a Two-Stage Analysis. Biometrics.

[47] Möhring J., Piepho H.P. (2009). Comparison of Weighting Methods in Two-Stage Analysis of Plant Breeding Trials. Crop Sci..

[48] Schrag T.A., Möhring J., Maurer H.P., Dhillon B.S., Melchinger A.E., Piepho H.P., Sørensen A.P., Frisch M. (2009). Molecular marker-based prediction of hybrid performance in maize using unbalanced data from multiple experiments with factorial crosses. Theor. Appl. Genet..

[49] Covarrubias-Pazaran G. (2016). Genome-Assisted Prediction of Quantitative Traits Using the R Package sommer. PLoS ONE.

[50] Bhatia R., Davis C. (2000). A Better Bound on the Variance. Am. Math. Mon..

[51] Falush D., Stephens M., Pritchard J.K. (2003). Inference of Population Structure Using Multilocus Genotype Data: Linked Loci and Correlated Allele Frequencies. Genetics.

[52] Gianola D., van Kaam J.B.C.H.M. (2008). Reproducing Kernel Hilbert Spaces Regression Methods for Genomic Assisted Prediction of Quantitative Traits. Genetics.

[53] Crossa J., Martini J.W., Gianola D., Pérez-Rodríguez P., Jarquin D., Juliana P., Montesinos-López O., Cuevas J. (2019). Deep Kernel and Deep Learning for Genome-Based Prediction of Single Traits in Multienvironment Breeding Trials. Front. Genet..

[54] Bernardo R. (2020). Reinventing quantitative genetics for plant breeding: Something old, something new, something borrowed, something BLUE. Heredity.

[55] Wilson S.E. (2023). Statistical Considerations for Applying Genomic Prediction to Potato. Ph.D. Thesis.

